# Supplementary material for: Daily Paced Breathing Sessions Induce Left Orbitofrontal Volume Changes Linked to Cognitive Outcomes
Source: medRxiv. 2026 Mar 4:2026.03.02.26347452. Preprint. [Version 1] doi: 10.64898/2026.03.02.26347452 (PMC13004101; doi:10.64898/2026.03.02.26347452)
Supplement: Supplement 1 [file media-1.pdf]

Supplementary Materials

Daily Paced Breathing Sessions Induce Left Orbitofrontal Volume Changes Linked to Cognitive Outcomes

Hyun Joo Yoo[1], Andy Jeesu Kim[1], Martin J. Dahl[1,2,3], Kalekirstos Alemu[1], Kaoru Nashiro[1], Christine Cho[1], Noah Mercer[1], Paul Choi[1], Hye Rynn, J. Lee[1], Jungwon Min[1], Nicole F. Rose[4], Julian F. Thayer[5], and Mara Mather[1,6,7]

Supplementary Figure S1

Overview of the Study Design and Analytical Framework Linking Training HRV, Cortical Plasticity, and Cognitive Performance

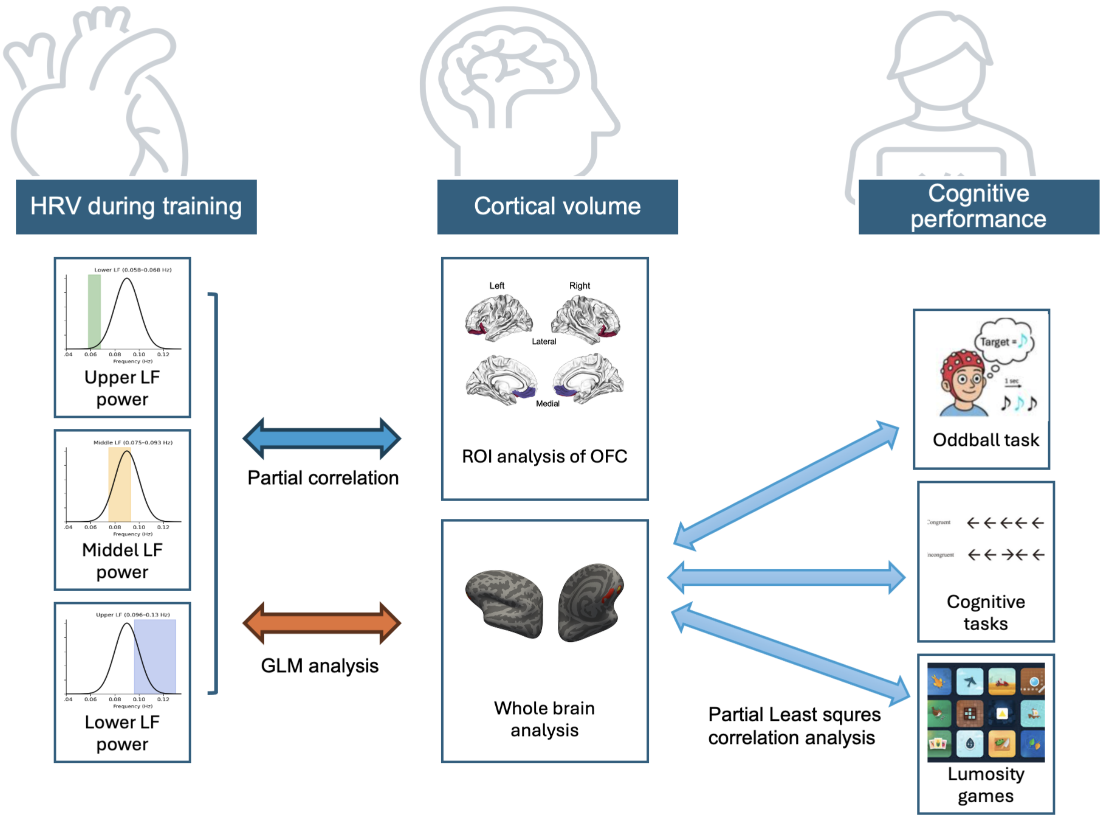

Note. The illustration of the auditory oddball task is adapted in part from (Kim et al., 2026). The Lumosity game image is reproduced from publicly available materials on the Lumosity website for illustrative purposes only.

Supplementary Figure S2

Overview of the 12-week study schedule

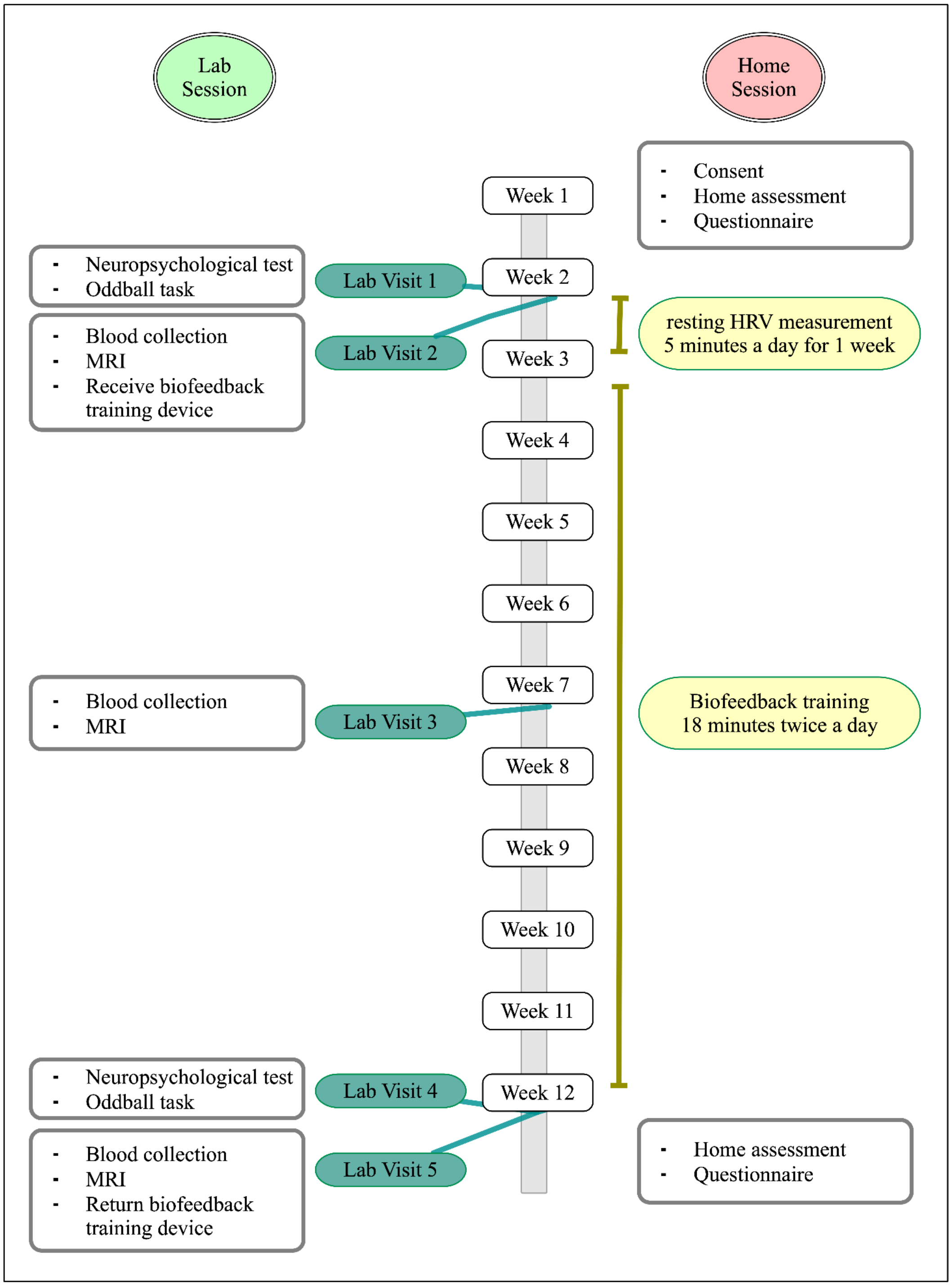

Supplementary Figure S3

Visual breathing pacer interface used during training

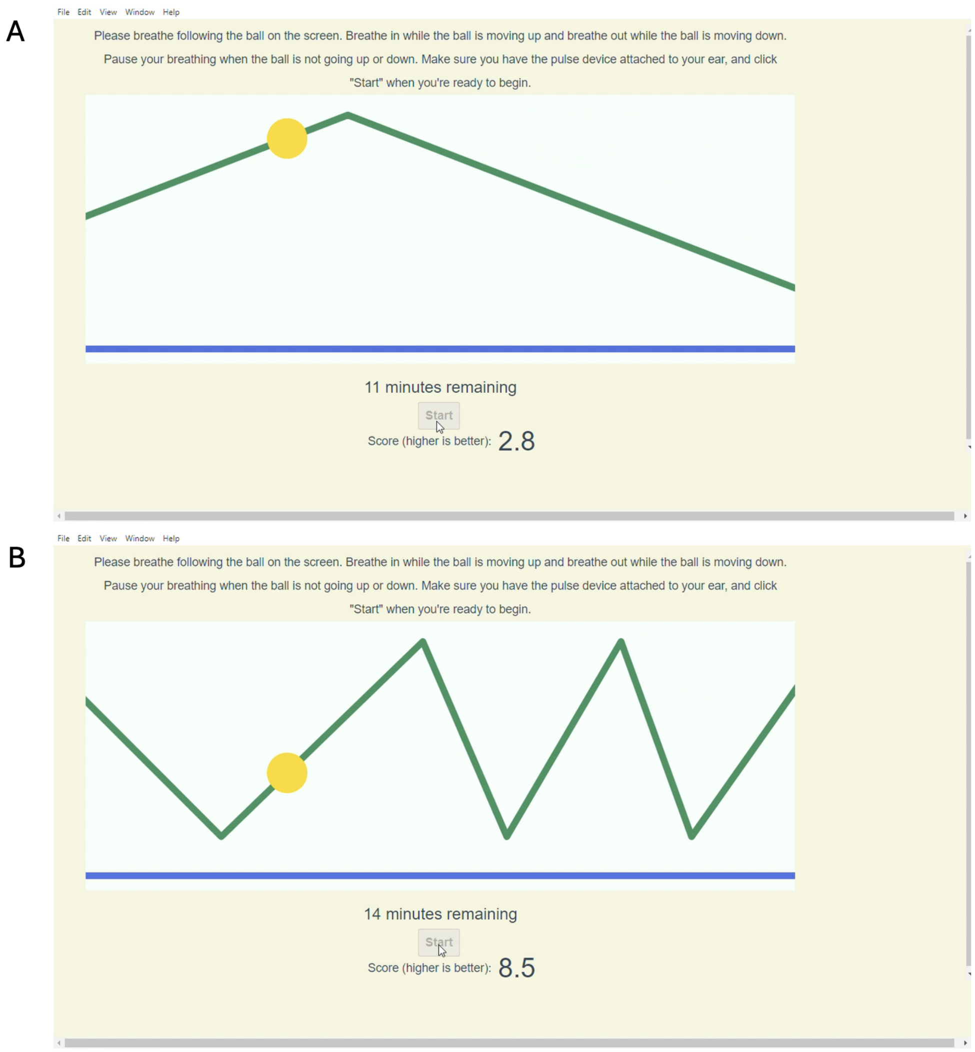

Note. Panels A and B show screenshots from the breathing training application used in the study. The application displayed a visual pacer consisting of a ball moving vertically along a line, which participants followed by inhaling as the ball moved upward and exhaling as it moved downward. Participants completed paced breathing segments according to their assigned condition (A: slow-paced breathing; B: random-paced breathing; see Methods).
During each segment, a condition-specific performance score was displayed below the pacer. In the slow-paced breathing condition, the score reflected the magnitude of heart rate oscillations, with higher values indicating greater oscillatory activity. In the random-paced breathing condition, the displayed score was inversely scaled such that higher values corresponded to reduced heart rate oscillations.

Supplementary Table S1

Schedule of home-based cognitive tasks administered during week 1 and week 12.

Type	Test	Day 1	Day 2	Day 3	Day 4	Day 5	Day 6
Cognition	CVLT Part 1					O	
	CVLT Part 2 (after 20 min interval)					O	
	Working memory (n-back)		O	O	O	O	O
	Face-name associative memory	O	O	O	O	O	O
	Pattern separation task - Part 1 (encoding)		O		O		O
	Pattern separation task - Part 2 (test)		O		O		O
	Task switching			O			
	Flanker			O		O	
	Spatial orientation	O	O	O	O		O
	Verbal Fluency	O	O	O	O		O

Note. The same sequence of tasks was administered during Week 2. Empty cells indicate that the task was not administered on that day.

Supplementary Table S2

Lumosity games

Set	Game name	Category	Description
1	Familiar faces	Memory	Participants play a waiter's role and earn higher tips if they can remember their customers' names and food orders
1	Tidal treasures	Memory	On each trial, participants are shown several unique ocean treasures and must choose one that they have not already selected in that round. Rounds can include up to 35 items, and some items are quite similar
1	Lost in migration	Attention	Participants indicate the direction of the central bird in the formation while ignoring the distractors around it
1	Splitting seeds	Attention	Participants evenly divide a pile of seeds without counting them
1	Pirate passage	Reasoning	Participants navigate their ship to reach the treasure island without colliding with other pirate ships
1	Ebb and flow	Flexibility	Participants view green or orange leaves moving across a pond and indicate the direction of where green leaves are pointing or where orange leaves are moving
2	Memory match	Memory	Participants must quickly determine whether a flashcard symbol matches the one presented two items ago
2	Word bubbles rising	Language	Participants generate as many words as possible that start with the same starting letters (e.g., res, medi) within the time limit
2	Raindrops	Math	Participants perform each math problem inside each raindrop before it reaches the bottom of the screen. Math problems include addition, subtraction, multiplication, and division
2	Penguin pursuit	Attention	Participants guide a penguin through a maze to reach a reward of fish at the end before the other penguin does. When the maze rotates, participants must rotate their mental map of the maze and recalibrate the directions to get to the goal
2	Brain shift	Flexibility	Participants are shown a letter-number pair (e.g., 5E) on the top or the bottom card. If the letter-number pair shows up on top, participants indicate whether the number is even; if it appears at the bottom, participants indicate whether the letter is a vowel or not
2	Color match 2	Flexibility	Participants indicate the color of a written word while ignoring the meaning of the word

Supplementary Table S3

Basic participant characteristics across conditions

	All	Slow-paced breathing	Random-paced breathing	Condition difference (t or χ^2)	Condition difference (p)
N	55	29	26		
Age	60.1 (5.99)	60.6 (5.58)	59.6 (6.48)	0.50	.553
Gender	29 F/26 M	15 F/14 M	14 F/12 M	0.02	.875
Education	16.6 (2.36)	15.9 (1.87)	17.4 (2.62)	-2.50	.016
MoCA	26.4 (2.24)	26.19 (2.25)	26.52 (2.25)	-0.54	.595
Race				1.99	.737
African American	6	2	4		
Asian	11	7	4		
Bi-racial	5	3	2		
Caucasian	30	16	14		
Other	0	0	0		
Prefer not to state	3	1	2		

*$p < 0.05$; **$p < 0.01$; ***$p < 0.001$, 2-tailed. Note: Condition differences were statistically tested using t-tests for age and education and chi-square tests for gender and race.
